# From registration to publication: A study on Dutch academic randomized controlled trials

**DOI:** 10.1002/jrsm.1379

**Published:** 2020-01-28

**Authors:** Joost Huiskens, Boudewijn R.J. Kool, Jean‐Michel Bakker, Emma R.J. Bruns, Stijn W. de Jonge, Pim B. Olthof, Belle V. van Rosmalen, Thomas M. van Gulik, Lotty Hooft, Cornelis J.A. Punt

**Affiliations:** ^1^ Department of Surgery, Amsterdam UMC, Location AMC University of Amsterdam Amsterdam The Netherlands; ^2^ Department of Medical Oncology, Amsterdam UMC, Location AMC University of Amsterdam Amsterdam The Netherlands; ^3^ Cochrane Netherlands, Julius Center for Health Sciences and Primary Care University Medical Center Utrecht Utrecht The Netherlands; ^4^ Department of Epidemiology Harvard T.H. Chan School of Public Health Boston Massachusetts; ^5^ Department of Surgery Erasmus Medical Center Rotterdam The Netherlands

## Abstract

**Introduction:**

Registration of clinical trials has been initiated in order to assess adherence of the reported results to the original trial protocol. This study aimed to investigate the publication rates, timely dissemination of results, and the prevalence of consistency in hypothesis, sample size, and primary endpoint of Dutch investigator‐initiated randomized controlled clinical trials (RCTs).

**Methods:**

All Dutch investigator‐initiated RCTs with a completion date between December 31, 2010, and January 1, 2012, and registered in the Trial Register of The Netherlands database were included. PubMed was searched for the publication of these RCT results until September 2016, and the time to the publication date was calculated. Consistency in hypothesis, sample size, and primary endpoint compared with the registry data were assessed.

**Results:**

The search resulted in a total of 168 Dutch investigator‐initiated RCTs. In September 2016, the results of 129 (77%) trials had been published, of which 50 (39%) within 2 years after completion of accrual. Consistency in hypothesis with the original protocol was observed in 108 (84%) RCTs; in 71 trials (55%), the planned sample size was reached; and 103 trials (80%) presented the original primary endpoint. Consistency in all three parameters was observed in 50 studies (39%).

**Conclusion:**

This study shows that approximately one out of four Dutch investigator‐initiated RCTs remains unpublished 5 years after initiation. The observed low overall consistency with the initial study outline is a matter of concern and warrants improvements in trial design and assessment of trial feasibility.

## 1 INTRODUCTION

The declaration of Helsinki (1964) is the cornerstone of modern human research ethics. Based on the fundamental principle of respect for the individual and the right to take informed decisions regarding participation in research, the declaration morally binds physicians and scientists to publish clinical trial information and results.^1^ It has been estimated that only half of the one million trials started since 1948 have been published.^2^

Patients who give informed consent to participate in scientific research and thereby agree to exposure to an experimental treatment do so under the assumption that they contribute to medical science. If investigators fail to publicly communicate these results, this contribution is nullified and the conditions for the initial agreement for participation are not met. This implies that invaluable information for the selection of optimal treatment and for the allocation of future research funds are withheld from the scientific community. This also results in loss and distortion of evidence, impairment of the practice of evidence‐based medicine, and a potential waste of funds on duplicative trials. Failure to publish research results has been considered as scientific misconduct.^3^, ^4^

The validity of clinical trial results starts with a carefully designed and conducted trial. Adherence to the trial protocol in the eventual trial report is essential in minimizing bias and prevention of selective reporting. Reporting of results based on outcomes or any specific interest of the investigator will increase the risk of bias and potentially hampers evidence‐based medicine. Unfortunately, discrepancies between a registered trial protocol and its publication are still frequently reported.^5^, ^6^, ^7^, ^8^

Since July 2005, the International Committee of Medical Journal Editors requires trials to be registered before the enrolment of the first patient in order to prevent selective publication of trial outcomes in an effort to reduce this form of publication bias.^9^ Besides the obligation to publish trial results, it is essential that these results become available within an appropriate period to ensure that clinical decisions can be made on the most recently available evidence. However, since 2005, several reports have shown that between 25% and 50% of the clinical trials experience significant delay or even remain unpublished.^8^, ^10^, ^11^, ^12^, ^13^, ^14^, ^15^, ^16^ The tendency to publish only positive results is just one of the reasons many trials remain unpublished.^17^

Even though academic medical centers are at the heart of clinical research, their publishing and reporting of results are not optimal.^14^, ^16^, ^18^, ^19^ In The Netherlands, there is an excellent track record of investigator‐initiated clinical research that is considered because of a well‐organized research infrastructure in which academic and general hospitals are actively participating.^20^

This study aims to investigate the rates of publication of trial results within 2 years after planned completion or premature closure of patient accrual. The prevalence of consistency in hypothesis, sample size, and primary endpoint between the registry and the corresponding publication of Dutch investigator‐initiated randomized controlled trials (RCTs) was investigated.

## 2 METHODS

In August 2016, information of all RCTs registered in the prospective Trial Registry in The Netherlands (NTR), which is part of the WHO primary registries, was collected. To ensure an adequate period allowing researchers to publish their results by August 2016 (ie, 5 years after data completion), only RCTs with a reported completion date between December 31, 2010, and January 1, 2012, were included. To identify all RCTs with a responsible party based at a Dutch academic medical center, the “SPONSOR/INITIATOR” field of the NTR was used. All RCTs that had one of the eight Dutch academic medical centers submitted in this field were selected for analysis. Multicenter and multinational trials were included only if a Dutch academic center had initiated the trial. Of every RCT the sample size, the study design (single or multicenter design), and the studied condition according to the http://clinicaltrials.gov categories were collected.

The outcome parameters included the number of published RCTs, the number of RCTs with published results within 2 years after completion of patient accrual, and the consistency between the trial registry data and published data that was scored in respect of the main hypothesis, sample size, and primary endpoint.

### 2.1 Search strategy to identify publication of RCTs

The PubMed service was used to search the biomedical literature for publication using the unique registration numbers of the RCTs between January 2011 and September 2016 by two reviewers (BK and JH). If no publication was identified, the search was expanded with details of the registered trial, such as author, acronym, primary outcome, scientific title, and hypotheses. Finally, if still no publication was found, the principal investigators of the study were contacted by email. A reminder was sent to every contact person that did not respond to the first email within 1 week. If no publication was found, and if the principal investigators did not reply to either email, it was assumed that the trial results had not been published.

The earliest publication of an RCT reporting the main results including the primary endpoint was selected. If multiple primary endpoints were registered, the earliest publication reporting at least one of the primary endpoints was used to assess the time to publication. All articles were retrieved by BK, and a second reviewer JH independently reviewed all selected articles. Any uncertainties were discussed until consensus was reached. In case BK was not able to identify the publication of a registered RCT, a second search was performed independently by JH. The time to publication curve was generated using the Kaplan‐Meier method.

### 2.2 Data selection of published trials

Full copies of all identified articles were obtained, and the time span in months between the completion date and the publication date was calculated using the “completion date” field of the NTR database and the publication date. For RCTs with an earlier online publication date (ePub date), the ePub date was used as the publication date. The following variables were collected from the available publications: the hypothesis, sample size, and the primary outcome.

### 2.3 Assessment of consistency: Comparison of publications with their protocol

All retrieved corresponding publications of registered RCTs were used for the consistency assessment. Consistency in hypothesis was assessed by comparing the primary hypothesis provided in the NTR with the hypothesis in the published article. When a hypothesis was not provided in the NTR, that RCT was recorded as discrepant in hypothesis for the analysis.

In case multiple primary endpoints were registered, an RCT was only considered consistent in primary endpoint if all primary endpoints were published and no new primary endpoints were provided in the publication. The primary endpoint was also considered discrepant if it was not reported in the NTR.

It is mandatory in the NTR to register the sample size of a trial. The sample size calculation was considered discrepant if the sample size calculation of the publication differed from the NTR. When no sample size calculation was provided in the publication, and the recruited number of patients did not differ more than 5% from the registered sample size, the trial was considered consistent in sample size calculation.

If a published hypothesis, primary endpoint, and/or sample size showed discrepancy with the information as registered in the NTR, but a transparent and clearly formulated explanation for the deviation was provided in the publication, the RCT was considered consistent on this issue.

## 3 RESULTS

Between December 31, 2010, and January 1, 2012, a total of 168 RCTs sponsored by a Dutch academic center were registered in the NTR. These 168 RCT had a total sample size of 55.821 patients (median 120, IQR 50‐264). Among the 168 RCTs, 67 (40%) had a multicenter design and 87 (52%) were planned to enroll more than 100 patients. Nutritional and metabolic disorders (18%), disorders in behavior (18%), cancer and other neoplasms (11%), and cardiovascular diseases (11%) were the most frequently studied conditions. Additional RCT characteristics are summarized in Table 1.

**Table 1:** Overall characteristics and dissemination of randomized controlled trials across Dutch academic centers (completion date between December 31, 2010, and January 1, 2012)

	Trials Registered N	Overall Rate of Publication N (%)	Median Time From Completion Date to Published Results in Months (IQR)	Rate of Results Published ≤24 months of Study Completion Date N (%)
Total	168	129 (77)	30 (19‐43)	50 (20)
Center				
I	29	24 (83)	29 (16‐41)	9 (31)
II	28	21 (75)	24 (21‐43)	11 (39)
III	16	15 (94)	22 (10‐45)	4 (25)
IV	24	17 (71)	25 (15‐48)	9 (38)
V	21	15 (71)	25 (15‐47)	7 (33)
VI	10	8 (80)	33 (22‐44)	2 (20)
VII	13	11 (85)	44 (27‐53)	2 (15)
VIII	27	18 (67)	32 (19‐42)	6 (22)
Study sites				
Multicenter	68	51 (75)	33 (18‐52)	18 (35)
Single center	100	78 (78)	29 (19‐40)	32 (41)
Number of enrolled patients				
≤100	80	62 (78)	30 (18‐46)	22 (35)
>100	88	67 (76)	31 (19‐43)	28 (42)
Conditions studied				
Nutritional and metabolic disorders	30	24 (80)	28 (15‐36)	11 (46)
Behavior disorders	30	23 (77)	25 (16‐36)	11 (48)
Cardiovascular diseases	19	14 (74)	29 (18‐34)	5 (36)
Cancer and other neoplasms	19	16 (84)	27 (21‐35)	7 (44)
Nervous system diseases	10	7 (70)	47 (19‐55)	3 (43)
Muscle, bone, and cartilage diseases	9	6 (67)	32 (12‐46)	2 (33)
Conditions of the urinary tract, sexual organs, and pregnancy	9	6 (67)	47 (33‐58)	0 (0)
Wounds and injuries	7	7 (100)	48 (39‐59)	1 (14)
Viral diseases	5	5 (100)	36 (25‐53)	1 (20)
Respiratory tract diseases	4	1 (25)	32 (32‐32)	0 (0)
Infectious diseases	4	2 (50)	15	1 (50)
Digestive system diseases	3	2 (67)	36	1 (50)
Others	19	16 (84)	33 (20‐45)	7 (44)

### 3.1 Publication of results

**Figure 1:**
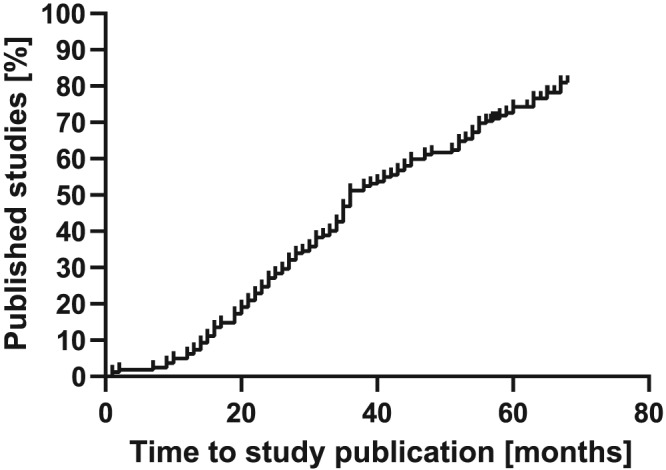
Kaplan‐Meier curve of publication rates of randomized controlled trials across Dutch academic centers (closing date 2011)

**Figure 2:**
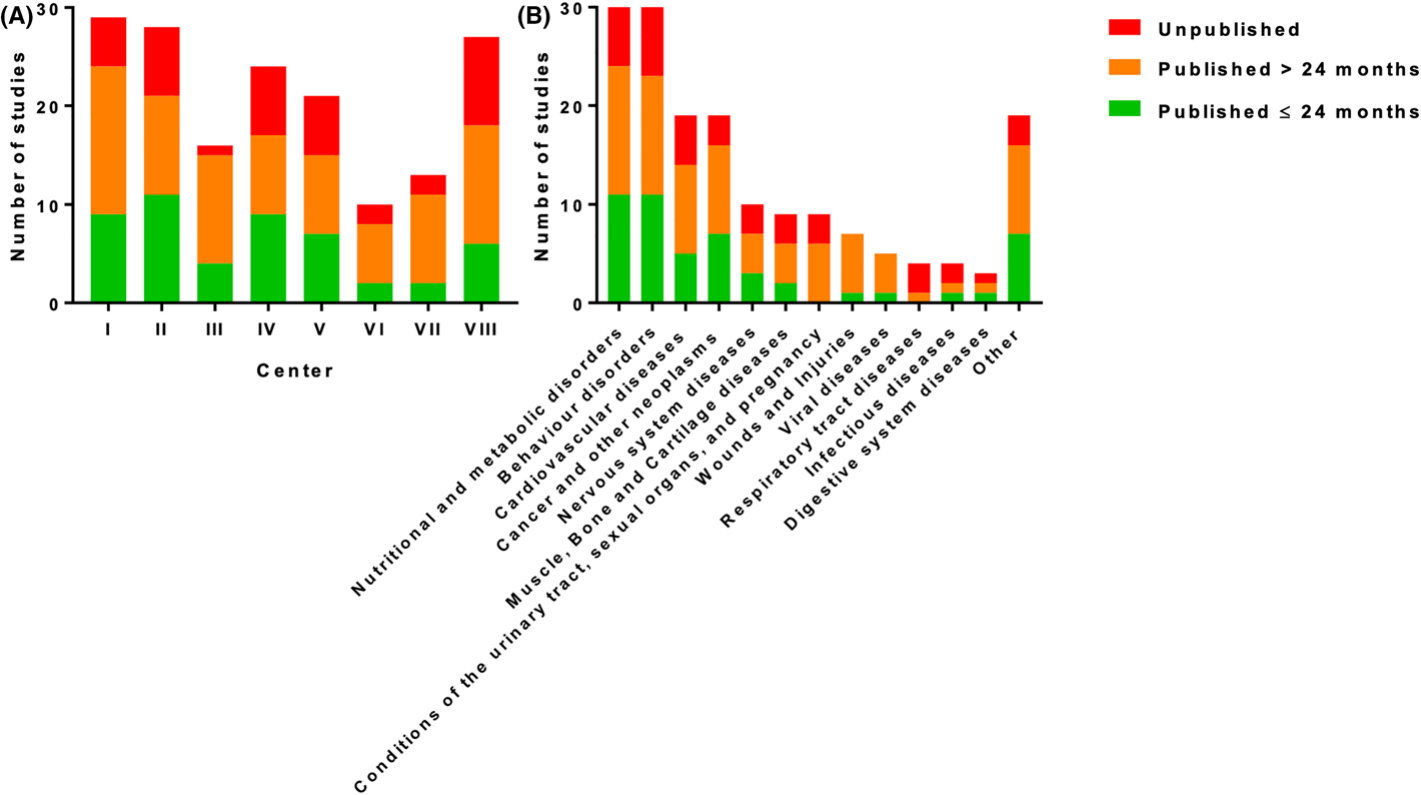
Time to publication of results for completed randomized controlled trials across (A) Dutch academic centers and (B) topics (closing date 2011) [Colour figure can be viewed at http://wileyonlinelibrary.com]

In total, 129 (77%) out of 168 RCTs were published in a medical journal as of October 2016 with a median time to publication of 30 months (IQR 19‐43). An overview of the publication rate of RTCs is shown in Figure 1. Publication rates varied between the leading academic centers, ranging from 67% to 94%. The rates of RCTs published within 24 months ranged between 20% and 39% (Table 1). In addition, the differences in time to publication between study topics were displayed in Figure 2.

A large variety in time between the completion and publication date of an RCT was observed. Results of 50 (30%) RCTs were published within 24 months, and results of 79 RCTs (47%) were published more than 24 months after the completion date. Results of five (4%) RCTs were published before their closing date.

Of the 39 (23%) RCTs that were not published, 21 principal investigators responded to our emails. Of one RCT, contact information could not be found in the NTR, which leaves 18 (11%) of the 168 RCTs without any information on publication. The principal investigators who replied to our email indicated that eight RCTs were never published because the RCT had been prematurely discontinued or had never been initiated. Of the six RCTs, it was indicated that the conduct had been delayed, and consequently, publication was delayed. Contacts of four RCTs responded that the manuscript of their RCT was rejected by journals for publication. One principal investigator was in the process of writing the manuscript, and one replied that his PhD student had left, and therefore, the RCT was never published.

### 3.2 Consistency in hypothesis, primary endpoint, and sample size

Consistency in all three parameters was observed in 50 (39%) of the 129 published RCTs. In 108 RCTs (84%), consistency in the main hypothesis was observed. In total, 10 RCTs did not report a hypothesis in the NTR and were assessed as discrepant. Consistency of the published RCT in the primary endpoint was observed in 103 RCTs (80%) and in sample size in 71 RCTs (55%). In six RCTs (5%), no sample size calculation was provided in the publication, but the number of recruited patients was within 5% range from the registered sample size. Additional details on consistency in hypothesis, sample size, and primary endpoint are summarized in Table 2.

**Table 2:** Consistency in hypothesis, sample size, and primary endpoint of randomized controlled trials across Dutch academic centers (closing date 2011)

	Trials Published N	Overall Consistency N (%)	Consistency in Hypothesis N (%)	Consistency in Sample Size N (%)	Consistency in Primary Endpoint N (%)	Discrepancy in 3 Objects (%)
Total	129	50 (39)	108 (84)	71 (55)	103 (80)	3 (2)
Center						
I	24	11 (46)	21 (88)	12 (50)	19 (79)	1 (4)
II	21	10 (48)	18 (86)	15 (71)	17 (81)	0 (0)
III	15	6 (40)	13 (87)	9 (60)	11 (73)	0 (0)
IV	17	3 (14)	14 (82)	6 (35)	14 (82)	0 (0)
V	15	7 (47)	12 (80)	9 (60)	13 (87)	0 (0)
VI	8	2 (25)	6 (75)	4 (50)	5 (63)	1 (13)
VII	11	6 (55)	10 (91)	6 (55)	11 (100)	0 (0)
VIII	18	5 (28)	14 (78)	10 (56)	13 (72)	1 (6)
Study sites						
Multicenter	51	24 (47)	43 (84)	33 (65)	42 (82)	0 (0)
Single center	78	26 (33)	65 (83)	38 (49)	61 (78)	3 (4)
Number of enrolled patients						
≤100	62	27 (44)	52 (84)	35 (56)	50 (81)	2 (3)
>100	67	23 (34)	56 (84)	36 (54)	53 (79)	1 (1)
Conditions studied						
Nutritional and metabolic disorders	24	12 (50)	21 (88)	13 (54)	19 (79)	2 (8)
Behavior disorders	23	4 (17)	18 (78)	7 (30)	16 (70)	0 (0)
Cardiovascular diseases	14	5 (36)	12 (86)	7 (50)	12 (86)	0 (0)
Cancer and other neoplasms	16	9 (56)	11 (69)	12 (75)	15 (94)	0 (0)
Nervous system diseases	7	4 (57)	6 (86)	5 (71)	6 (86)	0 (0)
Muscle, bone, and cartilage diseases	6	2 (33)	4 (67)	4 (67)	3 (50)	1 (17)
Conditions of the urinary tract, sexual organs, and pregnancy	6	2 (33)	4 (67)	5 (83)	4 (67)	0 (0)
Wounds and injuries	7	2 (29)	7 (100)	4 (57)	5 (71)	0 (0)
Viral diseases	5	3 (60)	5 (100)	3 (60)	5 (100)	0 (0)
Respiratory tract diseases	1	1 (100)	1 (100)	1 (100)	1 (100)	0 (0)
Infectious diseases	2	0 (0)	2 (100)	1 (50)	1 (50)	0 (0)
Digestive system diseases	2	1 (50)	2 (100)	2 (100)	1 (50)	0 (0)
Others	16	5 (31)	15 (94)	7 (44)	15 (94)	0 (0)

In 32 of the 58 RCTs that were discrepant in sample size calculation, the calculated sample size differed from the registered sample size without an explanation. In 26 of the 58 RCTs that were discrepant in sample size calculation, no sample size calculation was provided in the publication and the recruited number of patients differed more than 5% of the registered sample size.

Of the 129 reported RCTs, 57 (44%) recruited 90% or less than the registered planned sample size. Of these 57 trial reports, 29 (22%) did not report a clear explanation for this lower accrual.

**Figure 3:**
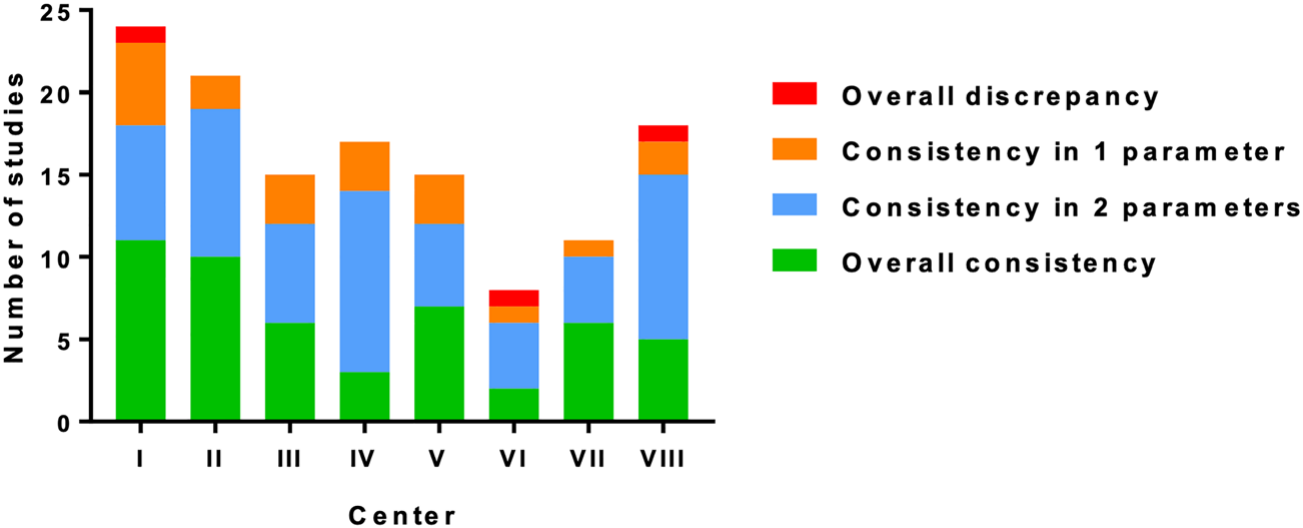
Rates of consistency in hypothesis, sample size, and primary endpoint of randomized controlled trials across Dutch academic centers (closing date 2011) [Colour figure can be viewed at http://wileyonlinelibrary.com]

Overall consistency as well as consistency in two, one, or even none of the parameters varied between academic centers ranging from 14% to 50% (Figure 3). Three RCTs demonstrated a discrepancy in all three parameters. There were some differences in consistency between topics, for instance, a low sample size consistency (30%) in RCTs on behavioral conditions.

The ideal trial registration is completed before the inclusion of the first patient. In this study, 68 (40%) out of the 168 trials had a registration date after the first inclusion date. There were no differences in trial characteristics between trials registered before or after the first inclusion and no differences in consistency parameters between the published trials.

## 4 DISCUSSION

A publication rate of 77% among 168 Dutch investigator‐initiated RCT within 5 years after the completion of patient accrual of the RCT was observed. Median time to publication was 30 months (IQR 19‐43), and only 30% (50/168) of the results were published within 2 years after the completion date. A low overall consistency in hypothesis, sample size calculation, and primary endpoint was found, with only 39% of the 129 published RCTs being consistent in all three parameters. Consistency of sample size reporting was observed in only 55% of the published RCTs.

The observed publication rate of Dutch investigator‐initiated RCT is higher than earlier reports.^8^, ^10^, ^11^, ^12^, ^13^, ^14^, ^15^, ^16^ However, in this study, we found that approximately one out of four Dutch RCTs remains unpublished after 5 years. It seems unlikely that these results will ever be made public. Investigators of these unpublished RCTs were planning to recruit a total of 8850 patients. Although the actual number of accrued patients in these unpublished RCTs is unknown, a significant number of patients will have been exposed to experimental treatments without any attribution to clinical science. This is in breach of the conditions to which agreement to participation by informed consent was met. Previous investigations have consistently shown that publication bias predominantly affects negative results.^21^, ^22^ There is evidence that nondisclosure of trial results and consequential distortion of evidence is harmful to patients.^23^ As an example, in the case of the use of antiarrhythmic drugs for secondary prevention of myocardial infarction, failure of timely publication of negative results has been estimated to have led to up to 75 000 preventable deaths a year in the United States alone.^24^ Timely reporting of results is essential to support evidence‐based decision making by clinicians and patients. Publishing results is also essential to allow more selective financing of trials and to prevent waste of funds by avoiding financing of duplicate trials that have proven to produce negative results in the past.

In the present study, the principal investigators reported several reasons for not publishing their results. The most common reasons for not publishing were that the RCT had not been started after trial registration or was prematurely discontinued, or that the conduct of the RCT was delayed. Slow patient recruitment is the most common reason for delay. Little evidence is available on strategies to improve recruitment to RCT.^25^ A realistic sample size calculation that incorporates the incidence of the studied condition as well as the amount of patients that actually qualify for the trial according to the envisaged inclusion criteria could help to generate a feasible trial protocol. In this respect, data on accrual of the same patient population in previous trials conducted in the same network would be supportive, since even with data on incidence, most investigators overestimate accrual. This implies that innovative tools are needed to improve recruitment. For this purpose, tools that use trial registers as a data repository could improve trial transparency and accrual.^26^ Another reason for not publishing was that finalized manuscripts were not accepted for publication by medical journals. This implies that journals contribute to publication bias, which is a known, longstanding but unsolved problem.^27^, ^28^ Publishing results is an ethical obligation of researchers and editors. Withholding results could have major consequences.^28^ Future studies could determine additional factor associated with the nonpublication of clinical trials such as effect size and statistical and clinical significance.

A potential solution could be to enable investigators to submit trial results to a trial register. In this way, regardless of publication of the manuscript, the trial results are accessible to the public. However, it is currently not possible to submit study results in the NTR other than in a plain text box. Another possible solution is that research ethical committees could have a more prominent role to ensure that trial results are published by monitoring the conduct of a trial.^29^ An obligation to register trials before data collection as a condition for ethical approval or funding could enhance both quantity and quality of registrations. This is especially important since the prospective registration of clinical trials may reduce research dissemination bias in clinical research.^30^

The incomplete consistency in hypothesis, sample size calculation, and primary endpoint are a continuous matter of concern.^6^, ^8^ Results of an RCT with discrepancy in hypothesis, sample size calculation, or primary endpoint might be unreliable and biased. Changes in trial protocol should be clearly reported and justified, as some may be well substantiated.

Our study has some limitations. Firstly, only RCTs that had a closing date in 2011 were selected. This period was chosen to provide a sufficient window to publish results while the relevance of results that are still not published after 5 years decreases rapidly. Additionally, only RCTs that were registered in the NTR, which is the primary register of The Netherlands, were included; however, Dutch RCTs may also have been registered in other registers such as http://clinicaltrials.gov or http://ISCRTN.com. Secondly, to find out whether an RCT was published, we ultimately contacted the principal investigators of whom only 51% responded, leaving 11% of the initial RCTs without any information on reasons for nonpublication. Finally, the delayed reporting of results may also be due to the publication strategy of the authors, with delays occurring after repeated rejection by journals, or due to the required follow‐up for the primary endpoint. However, when the required follow‐up is not reached, it can be debated whether the RCT is really closed and finished. Also, it cannot be excluded that the results of some unpublished studies were presented at conferences without a final publication. Although some might consider this sufficient, this is most often not sufficient to completely review all aspects of clinical trial results. Inversely, some clinical trials might be published but not registered at all. These are not captured in the current analysis, and therefore, the number of included trials could be underestimated.

Recently, two national initiatives were launched in order to facilitate researchers in the design, initiation, and conduct of clinical trials: the Dutch Clinical Research Foundation and Dutch Oncology Research Platform. The possibilities to share research expertise and establish collaborations should reduce the difficulties encountered in the conduct of clinical trials and help improve the timely publication of trial results.^31^

In conclusion, in a sample of 168 investigator‐initiated academic RCT, the results of 77% were published within 5 years. Although this is better than earlier reports, still one out of four RCTs remains unpublished. The observed low overall consistency is a matter of concern. Publication rates and consistency should be frequently studied to improve the conduction and reporting of RCTs. Solutions are warranted to improve the trial design, trial registration procedures, trial publication rates, and consistency between the trial register and publication of a manuscript.

## CONFLICT OF INTEREST

The author reported no conflict of interest.

## Data Availability

The data that support the findings of this study are available from the corresponding author upon reasonable request.
